# Divergent Conversion Efficiencies of *Mycobacterium* sp. 191574 for Various Phytosterols and Their Underlying Mechanisms

**DOI:** 10.3390/biom15111496

**Published:** 2025-10-23

**Authors:** Zifu Ni, Yingjing Bi, Zihao Wang, Yun Han, Yanlan Bi, Linshang Zhang, Shangde Sun

**Affiliations:** 1 College of Biological Engineering, Henan University of Technology, Zhengzhou 450001, China; 2 College of Food Science and Engineering, Henan University of Technology, Zhengzhou 450001, Chinashangdesun@haut.edu.cn (S.S.)

**Keywords:** *Mycobacterium* sp. 191574, steroid drugs, steroid-drug intermediates, biotransformation, proteomics, molecular docking

## Abstract

Steroid drugs have a broad range of applications in medicine. The microbial degradation of phytosterols for the synthesis of steroid drug intermediates holds significant potential for industrial applications. In this study, the transformation efficiency and underlying mechanisms of different phytosterols in *Mycobacterium* sp. 191574 were investigated. Among the tested compounds, β-sitosterol exhibited the highest conversion efficiency, followed by mixed sterols, while stigmasterol showed the lowest efficiency. Proteomic analysis identified key enzymes involved in sterol metabolism. Further molecular docking experiments revealed that acyl-CoA synthetase (A0A0T1W1C0) and hydrolase (A0A0T1W815) may act as rate-limiting enzymes contributing to the low conversion rate of stigmasterol. Significant differences in hydrogen bonding patterns and three-dimensional spatial structures between these enzymes and sterol-derived ligands were observed, resulting in reduced binding affinity of stigmasterol. This study provides a theoretical basis for the optimization of sterol biotransformation processes and offers a foundation for improving the biological production efficiency of sterol-based pharmaceutical intermediates.

## 1. Introduction

Steroids are a class of non-nutritive, lipophilic compounds characterized by a common cyclopentane polyhydrophenanthrene core structure. They are widely distributed in plants, animals, and fungi, and exhibit a diverse range of physiological activities [1]. These compounds primarily form hormone-receptor complexes by binding to specific intracellular receptors and function as multi-domain, ligand-dependent transcriptional regulators in the nucleus [2]. Through this mechanism, they can modulate the expression of hundreds to thousands of target genes, thereby influencing protein synthesis [3]. Consequently, steroids have significant applications in the development of anti-inflammatory, immunosuppressive, hormone replacement, and other therapeutic drugs [2]. The biological activity of steroids is closely related to their chemical structure; various modification reactions such as oxidation, reduction, and hydrolysis can significantly affect their biological activity and biocompatibility [4]. Currently, steroids have become the second largest class of drugs globally after antibiotics, with an annual production value of approximately USD 13.5 billion and a global output exceeding 1 million tons. Over 400 steroid drugs are authorized and in circulation, playing a crucial role in the pharmaceutical industry [5].

Although sterols are widely present in organisms, their low abundance and structural diversity pose challenges for drug discovery and development. Currently, the synthesis of steroidal compounds can be achieved either through complex total synthesis routes, starting from simple organic small molecules such as cyclohexanone and terpenoids and constructing a complete steroidal core via dozens of steps, or through semi-synthetic approaches, which involve limited chemical modifications of natural sterols such as diosgenin from yams root and hecogenin from sisal [4,6]. However, these processes are often complex, generate by-products, and face other issues, with most syntheses remaining at the laboratory scale, thus failing to meet industrial production requirements. The rapid advancement of biological information has provided strong support for exploring and transforming sterol biosynthesis pathways. Among these, microbial endogenous transformation pathways for sterol synthesis and modification have become a research hotspot. The discovery by Sih et al. that actinomycetes can remove the side chain from saturated sterols and convert them into androstenedione has stimulated increased interest among researchers in using biotransformation to produce C19 and C22 steroid drug intermediates [7]. Biotransformation-based methods for producing steroidal drug intermediates involve modifying unsaturated bonds in the side chain or ring structures based on the unique multi-ring skeleton of steroidal compounds [8]. The key raw material in this process is phytosterol, which is structurally similar to steroidal drugs and serves as a natural precursor substrate for steroid drug synthesis.

**Figure 1:**
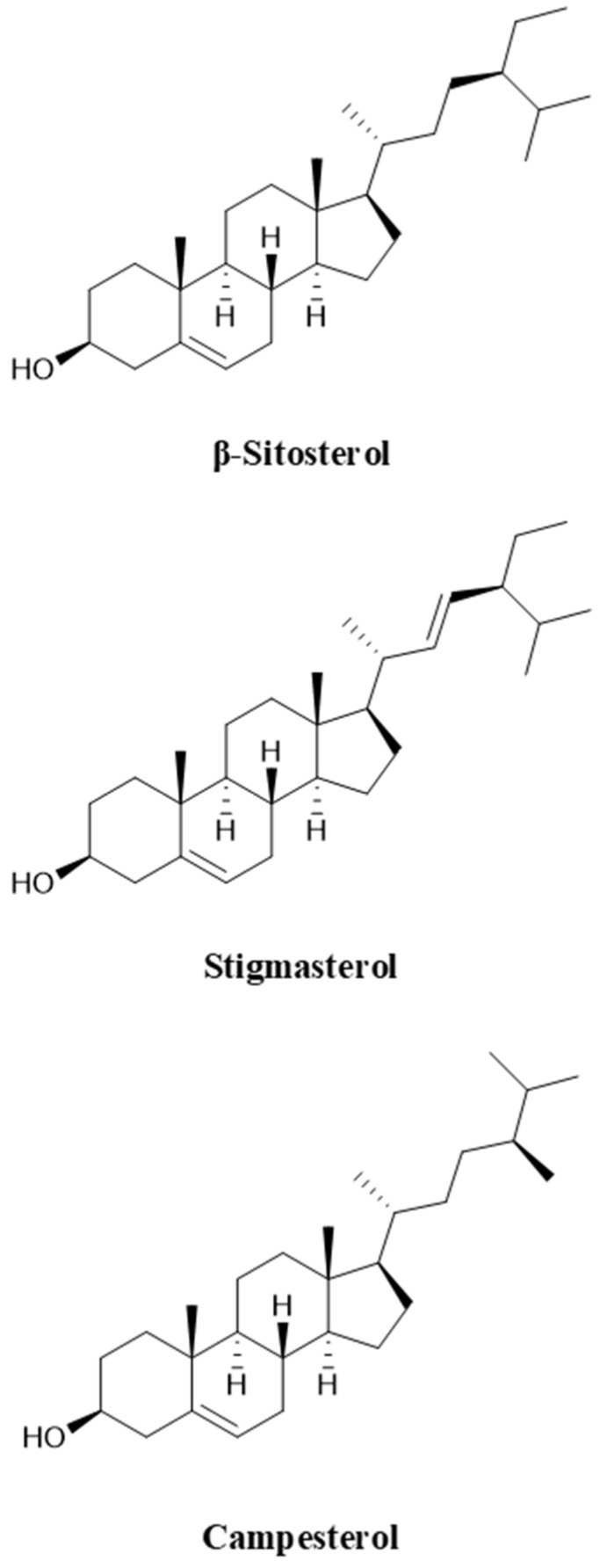
Molecular structures of β-sitosterol, stigmasterol and campesterol.

Unlike the higher fatty acid ester forms of sterols in animals, phytosterols in plants occur in the form of free sterols, sterol esters, sterol glycosides, and acyl sterol glycosides [9]. Among them, β-sitosterol, stigmasterol, and campesterol are the most common free sterols, which are widely derived from oil crops such as corn and soybean, accounting for more than 70% of phytosterol production, and their molecular structures are shown in Figure 1 [10]. The primary structural difference lies in the presence or absence of a double bond between C20 and C21 and the nature of the side chain at C22. Specifically, stigmasterol possesses a double bond at C20–C21, whereas β-sitosterol and campesterol contain single bonds at this position. In terms of side chains, both β-sitosterol and stigmasterol are linked to an ethyl group at C22, while campesterol carries a methyl group. These structural variations result in significant differences in the side chains of different sterols. In biotransformation studies, mixed sterols are commonly employed as fermentation substrates, primarily because the separation and purification of individual sterols involve costly refining processes [11]. Using sterol mixtures as substrates reduces the requirement for high purity and thus offers a more economical approach. For example, Li et al. [12] utilized a sterol blend comprising β-sitosterol, stigmasterol, and campesterol. García-Fernández et al. [13] employed a mixture of cholesterol, β-sitosterol, campesterol, and stigmasterol with gradually decreasing concentrations. Zhou et al. [11] investigated a substrate composition consisting of 51.7% β-sitosterol, 27.2% stigmasterol, 17.1% campesterol, and 4.0% brassicasterol. Similarly, Xiao et al. [14] adopted a blend containing 45.0% β-sitosterol, 26.2% stigmasterol, 23.5% campesterol, and 3.2% brassicasterol. Notably, the type and proportion of sterols in different fermentation substrates vary considerably, leading to distinct differences in microbial conversion efficiency. These variations directly influence the physicochemical properties and biological activities of sterols following biotransformation [15]. Therefore, the composition of sterol substrates is a critical factor in determining both the efficiency of microbial transformation and the functional characteristics of the resulting products. Careful selection and optimization of substrate composition will thus be essential for improving the industrial application of sterol biotransformation.

At present, microorganisms identified for sterol conversion include *Arthrobacter*, *Mycobacterium*, *Bacillus*, and *Streptomyces* [16,17,18]. Among these, *Mycobacterium* species have been the most extensively studied due to their strong sterol-degrading capacity, although they are difficult to cultivate and growing very slowly, they still show obvious advantages in sterol degradation [17]. For instance, molecular modification of *Mycobacterium neoaurum* to overexpress NADH dehydrogenase improved cell viability by 54.17%, increased the phytosterol conversion rate from 23.92% to 94.98%, and enhanced androstenedione (AD) production by 12.75-fold [19]. In addition, genetic engineering of the *M. neoaurum* LY-2 strain specifically through knockout of the KasB gene and overexpression of the pcc, hsd4a, and vgb genes, enabled androst-1,4-diene-3,17-dione (ADD) production of up to 11.12 g/L [20]. These findings highlight the potential of metabolic pathway engineering to reprogram microbial biosynthesis of steroid intermediates. However, wild-type strains tend to further degrade the steroid nucleus after cleaving the C17 side chain of sterols, thereby preventing the accumulation of valuable C19 and C22 steroidal drug intermediates [21]. To overcome this limitation, wild strains have been optimized through mutation breeding, key enzyme engineering, and related strategies to better meet the demands of industrial-scale production [22,23,24]. Precise regulation of key metabolic nodes is urgently needed to enhance the selective synthesis of specific steroid drug intermediates and improve the overall efficiency of microbial transformation processes.

The development of genome sequencing technology has greatly facilitated the functional analysis of genes involved in phytosterol degradation in *Mycobacterium*, and most of the genes in the phytosterol metabolic pathway have now been elucidated [25]. This progress provides a solid foundation for the modification of biosynthetic pathways toward steroid drug intermediates. However, phytosterol metabolism is inherently complex, involving multiple metabolic routes and key enzyme systems in *Mycobacterium*. Therefore, it is of considerable significance to investigate the differences in conversion efficiency and the underlying mechanisms associated with different sterol types in these microorganisms. To address this issue, we selected the most abundant sterol monomers, β-sitosterol and stigmasterol, as model substrates. We systematically compared their overall conversion rates in *Mycobacterium* and employed integrated proteomics and molecular docking analyses to elucidate the molecular mechanisms driving the observed differences in conversion efficiency. This multi-scale approach provides valuable theoretical guidance for the optimization of sterol biotransformation in industrial fermentation processes.

## 2. Materials and Methods

### 2.1. Materials

AD and ADD were sourced from Macklin Biochemical Co., Ltd. (Shanghai, China). 2-Hydroxypropyl-β-cyclodextrin (HP-β-CD) and phytosterols, including β-sitosterol (93.11% purity, containing 6.89% campesterol), stigmasterol (95.79% purity, containing 3.54% β-sitosterol and 0.68% campesterol), and mixed sterols (β-sitosterol 60.70%, stigmasterol 22.41%, campesterol 16.79%) were obtained from the same supplier (Macklin). Nutrient broth medium was purchased from Beijing Auboxing Bio-Tech Co., Ltd. (Beijing, China). Citric acid, MgSO_4_·7H_2_O, K_2_HPO_4_, and (NH_4_)_2_HPO_4_ were supplied by Tianjin Zhiyuan Chemical Reagent Co., Ltd. (Tianjin, China). Methanol, acetonitrile, and ethyl acetate were procured from Tianjin Comio Chemical Reagent Co., Ltd. (Tianjin, China).

### 2.2. Strain and Cultivation

The bacterial strain *Mycobacterium* sp. 191574 was obtained from Beijing Beina Chuanglian Biotechnology Research Institute. Cultures were maintained on nutrient broth-agar slant medium (containing peptone 10.0 g/L, NaCl 5.0 g/L, beef extract powder 3.0 g/L, and agar powder 2.0 g/L) in 45 mL screw-cap tubes (8 mL medium/tube). Slants were sterilized by autoclaving at 121 °C for 20 min and stored at 4 °C. Colonies from slants were inoculated into 100 mL of nutrient broth medium in 250 mL flasks. Cultures were incubated at 30 °C with shaking (160 rpm) until OD_600_ nm reached 0.80–1.00.

Seed culture was inoculated (1% *v*/*v*) into fermentation medium and incubated at 30 °C with shaking (160 rpm) for 5 days. Fermentation medium: Citric acid: 2 g/L; (NH_4_)_2_HPO_4_: 3.75 g/L; HP-β-CD: 15 g/L; MgSO_4_: 1.5 g/L; K_2_HPO_4_: 0.5 g/L; Initial pH: 6.0. This fermentation medium was optimized to ensure optimal fermentation conditions for *Mycobacterium* sp. 191574 [26]. Carbon sources: each of β-sitosterol (1.23 g/L), stigmasterol (1.10 g/L), mixed sterols (1.19 g/L). Cultures (50 mL in 250 mL flasks) were sampled every 12 h. Conversion rates of sterols by *Mycobacterium* sp. 191574 were determined. Data were fitted to a five-parameter logistic (5PL) model using Origin 2021 software. Rate differences were assessed based on exponential growth phase slopes.

### 2.3. Fermentation Product Analysis

Fermentation broth was centrifuged at 8000× *g* for 3 min. The supernatant was transferred to a 250 mL separatory funnel and extracted three times with ethyl acetate. The combined organic phases were concentrated by rotary evaporation at 55 °C under reduced pressure. The residue was extracted by acetonitrile for five cycles (3 mL per cycles). The supernatant was collected and adjusted to a final volume of 25 mL, then filtered through a 0.22 μm membrane into HPLC vials for analysis.

Fermentation product was analyzed by our previous established HPLC method. Fuli LC5090 HPLC system (Fuli Analytical Instrument Co., Ltd., Zhejiang, China) was employed with a column of ZORBAX Eclipse Plus Phenyl-Hexyl (4.6 × 250 mm, 5 μm, Agilent Technologies, Inc., California, USA). The detection conditions of the product were as follows: column temperature 30 °C, detection wavelength 254 nm, elution with a 70% methanol solution at a flow rate of 1 mL/min, and injection volume of 10 μL. The retention time of AD is 8.96 min. Other detailed parameters can be found in our previous publication [26].

The conversion rate was calculated according to Formula (1).

(1) $$Conversion\;rate\left(\%\right)=\frac{n_{in}}{n_{st}}\times 100\%$$

(2) $$n_{in}=\frac{m_{ADD}}{M_{ADD}}+\frac{m_{AD}}{M_{AD}}+\frac{m_{1,4-HBC}}{M_{1,4-HBC}}+\frac{m_{4-HBC}}{M_{4-HBC}}\;$$

(3) $$n_{st}=\frac{m_{st}}{M_{st}}\;$$

where n_in_ represents the amount of intermediate, mol; n_st_ denotes the amount of sterol, mol; m stands for weight of sterol esters or intermediates, g; M indicates the molar weight, g/mol [27].

### 2.4. Proteomics

Proteomics were performed by Wuhan Maiwei Metabolism Biotechnology Co., Ltd. (Wuhan, China). Samples were lysed in buffer (1.5% SDS/100 mmol/L Tris-HCl) and centrifuged. Proteins in the supernatant were precipitated using acetone, then redissolved in 8 mol/L urea/100 mmol/L Tris-HCl solution. Reduction was carried out with dithiothreitol at 37 °C for 1 h, followed by alkylation with iodoacetamide in the dark at room temperature to block thiol groups. Protein concentration was determined using the Bradford assay. After reduction and alkylation, samples were diluted with 100 mmol/L Tris-HCl to reduce urea concentration below 2 mol/L. Trypsin was added at a 1:50 (*w*/*w*) enzyme-to-protein ratio for overnight digestion at 37 °C with shaking. The reaction was terminated with trifluoroacetic acid the next day. The supernatant was desalted using Sep-Pak C18 cartridges, dried under vacuum, and stored at −20 °C until analysis. MaxQuant v1.6.3.3 was used to search the UniProt species database. The FDR of peptide and protein levels were set to ≤1%, and only proteins with ≥2 unique peptides were identified. LFQ intensity was normalized by total peptide amount, and log2 conversion was performed. Missing values were interpolated according to normal distribution. The difference was tested by one-way ANOVA and corrected by Benjamini–Hochberg method. The significance threshold was set as adjusted *p* < 0.05, and the difference multiple threshold |log2FC| ≥ 0.58. GO and KEGG enrichment analysis showed that FDR ≤ 0.05 was significant, and key proteins were verified by Western blot.

LC-MS/MS analysis was performed on an Orbitrap Exploris 480 mass spectrometer coupled to an EASY-nLC 1200 nanoflow UHPLC system (Thermo Fisher Scientific Inc., Cleveland, OH, USA ). Separation used a C18 analytical column (75 μm × 25 cm, 1.9 μm particles) with mobile phases: A: 0.1% formic acid in water; B: 0.1% formic acid in 80% acetonitrile. A flow rate of 300 nL/min was maintained. Data were acquired in data-independent acquisition mode.

### 2.5. Molecular Docking

Starting from the amino acid sequence of the target protein, the three-dimensional structure of the protein was constructed by AlfaFold 3, the quality of the protein structure was detected by SAVES v6.0 (https://saves.mbi.ucla.edu/, accessed on 4 June 2025), and the non-conforming residues were re-revised by YASARA 20.12.24 software. Chemdraw 3D 17.0 software was used to draw the structure of small molecules and optimize it to the minimum energy. AutoDock Vina 1.1.2 was used to dock the enzyme with the substrate. The active center of the enzyme Gridbox was adjusted according to the size of the enzyme protein molecule. The number of dockings was 9, and other parameters were default. PyMOL 3.0 was used to visualize and analyze the modeled structures.

### 2.6. Data Processing

All experiments were performed in at least three independent replicates. Data were processed using SPSS 26.0 and Origin 2021 (Origin Lab). Results are expressed as mean ± standard deviation. Statistical significance was assessed by one-way analysis of variance (ANOVA), with *p* < 0.05 indicating significant differences. Post hoc pairwise comparisons were conducted using Duncan’s multiple range test.

## 3. Results and Discussion

### 3.1. Accumulation Rate of Total Yield of Different Steroid Drug Intermediates

**Figure 2:**
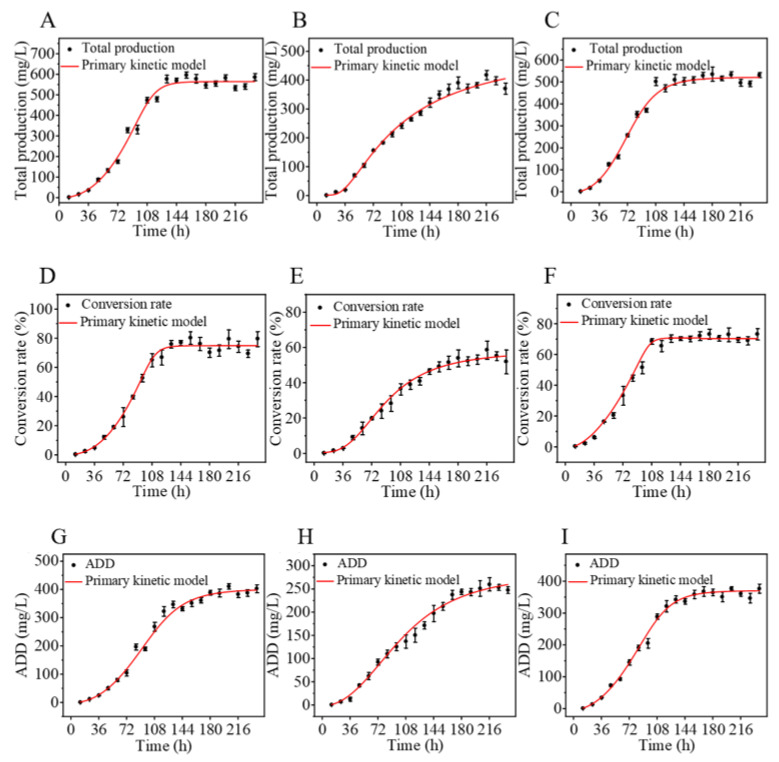
Kinetic profiles of β-sitosterol (**A**,**D**,**G**), stigmasterol (**B**,**E**,**H**), and mixed sterols (**C**,**F**,**I**). (**A**–**C**) kinetic profiles of total steroidal intermediate production; (**D**–**F**), kinetic profiles of sterol conversion rate; (**G**–**I**), kinetic profiles of ADD production.

Steroid drug intermediates are precursors used in the synthesis of steroid drugs and are mainly classified into C19 and C22 types [7]. C19 steroid drug intermediates primarily include AD and its dehydrogenation product ADD (Figure S1), as well as the hydroxylation product 9-hydroxy-4-androstene-3,17-dione (9α-OH-AD). AD is a precursor for the synthesis of male hormones, ADD can be used to synthesize progesterone and certain female hormones, and 9α-OH-AD serves as a precursor for the synthesis of glucocorticoids such as dexamethasone [28]. C22 steroid drug intermediates include 22-hydroxy-23,24-bisnorchol-4-ene-3-one (4-HBC) and its dehydrogenation product 22-Hydroxy-23,24-bisnorchol-1,4-diene-3-one (1,4-HBC) (Figure S1), which are commonly employed in the synthesis of progesterone and adrenocortical hormones [24]. Among these, AD and ADD have been developed as important precursors for a variety of steroid hormone drugs, including immunosuppressants, sex hormones, and neurosteroids. Phytosterols generally produce the same C19 and C22 degradation products during degradation by *Mycobacterium*, which is determined by their structural commonality, the conservation of metabolic pathways and the specificity of key enzymes. *Mycobacterium* accumulate a large amount of ADD after transporting phytosterols into the cells through an active transport system, which is consistent with our previous report [26]. Using β-sitosterol, stigmasterol and mixed sterols as substrates, the total yield of drug intermediates after transformation by mycobacteria was nonlinearly fitted. The fitting results are presented in Figure 2, and the corresponding first-order kinetic equations are listed in Table 1. Comprehensive analysis showed that the conversion efficiency of *Mycobacterium* varied with different sterol substrates. The conversion of β-sitosterol, stigmasterol and mixed sterol reached the maximum at 156, 180 and 180 h, respectively, with corresponding concentrations of sterol drug intermediates of 596.65, 391.09, and 535.80 mg/L. Their slopes of exponential growth phase were 11.01, 1.29 and 6.88, respectively. These results indicate that, under identical conditions, the conversion rate of β-sitosterol was the fastest, followed by mixed sterol, whereas stigmasterol exhibited the lowest conversion rate.

**Table 1:** First-order kinetics equation of divergent sterols.

		First-Order Kinetics Equation	Slope	R^2^
Total intermediate production	β-sitosterol	$y={-9.90+\left[563.32÷\left(1+{\left(\frac{113.71}{x}\right)}^{11.01}\right)\right]}^{0.21}$	11.01	0.9965
	stigmasterol	$y={1.73+\left[528.40÷\left(1+{\left(\frac{2.38}{x}\right)}^{1.29}\right)\right]}^{104.27}$	1.29	0.9969
	mixed sterols	$y={-3.29+\left[373.71÷\left(1+{\left(\frac{116.59}{x}\right)}^{6.88}\right)\right]}^{0.28}$	6.88	0.9985
Conversion rate	β-sitosterol	$y={0.88+\left[74.79÷\left(1+{\left(\frac{110.39}{x}\right)}^{15.41}\right)\right]}^{0.15}$	15.41	0.9976
	stigmasterol	$y={0.43+\left[57.95÷\left(1+{\left(\frac{82.81}{x}\right)}^{2.84}\right)\right]}^{1.21}$	2.84	0.9948
	mixed sterols	$y={-0.84+\left[71.63÷\left(1+{\left(\frac{105.20}{x}\right)}^{19.31}\right)\right]}^{0.09}$	19.31	0.9976
ADD production	β-sitosterol	$y={-1.34+\left[403.36÷\left(1+{\left(\frac{127.16}{x}\right)}^{5.59}\right)\right]}^{0.38}$	5.59	0.9971
	stigmasterol	$y={-1.69+\left[288.94÷\left(1+{\left(\frac{119.59}{x}\right)}^{2.77}\right)\right]}^{0.75}$	2.77	0.9973
	mixed sterols	$y={-3.29+\left[373.71÷\left(1+{\left(\frac{116.59}{x}\right)}^{6.68}\right)\right]}^{0.28}$	6.68	0.9958

### 3.2. Comparison of Conversion Rate of Different Sterols

Nonlinear fitting of sterol conversion data yielded the kinetic curves shown in Figure 2D–F, with the corresponding first-order kinetic equations presented in Table 1. The maximum conversion efficiencies of *Mycobacterium* sp. 191574 were 80.45% for β-sitosterol, 58.72% for stigmasterol, and 73.34% for mixed sterols. The slopes of the exponential growth phase were 15.41, 2.84, and 19.31 for β-sitosterol, stigmasterol, and mixed sterols, respectively. The accelerated conversion of the mixed sterols, surpassing that of both β-sitosterol and stigmasterol, can be attributed to the presence of campesterol (16.79%) in the mixture. Unlike stigmasterol, which contains a C22 double bond in its side chain, campesterol possesses a saturated alkyl side chain similar to β-sitosterol but with one fewer methyl group. This structural simplification reduces steric hindrance during enzymatic side-chain cleavage, thereby enhancing substrate accessibility and metabolic flux through the degradation pathway. Consequently, the inclusion of campesterol synergistically improved the overall conversion kinetics of the mixed sterol system. In addition, due to the hydrophobic nature of sterols, their biotransformation by *Mycobacterium* is also influenced by the cell wall structure and transport system [29]. Thus, modifying the structure and permeability of the *Mycobacterium* cell wall, as well as enhancing the activity of key enzymes involved in sterol uptake via active transport, are critical strategies for further improving sterol conversion efficiency [30].

### 3.3. ADD Accumulation Rate of Different Sterol Conversion Products

ADD is the product of complete degradation of the sterol C17 side chain, which is accomplished during the second round of β-oxidation of the sterol side chain. If the C17 side chain is not fully oxidized, the final accumulated products are the C22 steroid drug intermediates 4-HBC and 1,4-HBC [31]. As shown in Figure 2G–I, the accumulation of the main product ADD during the conversion of the three sterols was fitted using a nonlinear model, and the corresponding first-order kinetic equations of ADD yield are summarized in Table 1. Kinetic analysis of ADD accumulation showed that the yield from mixed sterols was the highest, followed by β-sitosterol, whereas stigmasterol exhibited the slowest accumulation. The slopes of the exponential growth phase were 6.68 for mixed sterols, 5.59 for β-sitosterol, and 2.77 for stigmasterol. During sterol biotransformation, side-chain degradation and nucleus oxidation are independent catalytic processes, and their sequence varies among microorganisms [9]. Among these reactions, 3-ketosteroid-Δ^1^-dehydrogenase (KstD) specifically catalyzes the oxidative dehydrogenation at C1,2 to form a double bond, thereby converting AD into ADD [32]. Thus, the high accumulation of ADD is closely associated with KstD activity. Knockout of the kstD1 gene in *Mycobacterium* directly results in the accumulation of AD, whereas enhancing the activity of KstD1 through molecular modification can significantly improve the catalytic efficiency toward substrates such as AD [31].

### 3.4. Proteomics Identification of Enzymes in Sterol Metabolic Pathway

**Figure 3:**
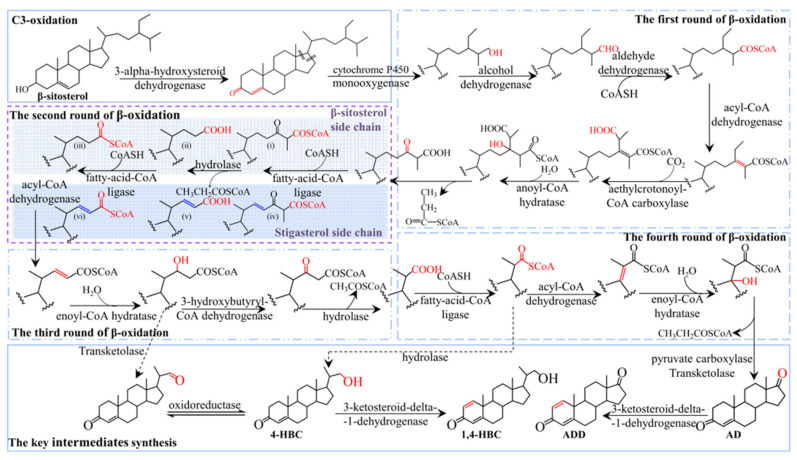
Pathway of sterol intermediates biosynthesis in *Mycobacterium*. Different frames represent different reaction processes, in which the purple frame represents the second cycle of β-oxidation. The light blue shaded area highlights the β-sitosterol conversion pathway, and the dark blue shaded area represents the predicted stigmasterol conversion pathway.

The above results indicated that *Mycobacterium* sp. 191574 exhibited significantly lower conversion efficiency for stigmasterol than for β-sitosterol, likely due to the C22-double bond in stigmasterol’s side chain. To elucidate the underlying limiting factors, proteomic analysis was employed. Proteomic profiling was performed using DIA-NN (v1.8.1) for protein identification and quantification, with a reference database of 5996 protein sequences. This identified 2463 peptides and quantified 724 proteins. Key enzymes implicated in sterol metabolism are detailed in Supplementary Table S1, including: C3-oxidation: One *3α-hydroxysteroid dehydrogenase* (3α-HSD); C17 side-chain initial cleavage: One *cytochrome P450 monooxygenase* and seven *monooxygenases* (including one *steroid-C27-monooxygenase*). Side-chain degradation: one *acetyl-CoA synthetase*, two *acetyltransferases*, seven *acyl-CoA synthetases*, sixteen *acyl-CoA dehydrogenases*, nine *enoyl-CoA hydratases*, seven *short-chain dehydrogenases*, five *oxidoreductases*, one *acyl-CoA thioesterase*, one *pyruvate carboxylase*, one *transketolase*. Steroid nucleus modification: one *3-ketosteroid-Δ*^1^*-dehydrogenase* and one *3-ketosteroid-9α-hydroxylase*. Comparative analysis between 60 h and 120 h revealed no significant temporal variations in enzyme expression. Integrating these proteomic findings with prior studies by Szentirmai and Wang, we propose the comprehensive sterol metabolic pathway illustrated in Figure 3 [18,19].

As illustrated in Figure 3, intracellular β-sitosterol undergoes C3 oxidation catalyzed by 3α-hydroxysteroid dehydrogenase, converting the C3-hydroxy group to a keto group while inducing isomerization of the Δ^5^ bond to Δ^4^. Side-chain degradation initiates via monooxygenase-mediated oxidation, followed by sequential β-oxidation cycles: Cycle 1: Alcohol dehydrogenase, aldehyde dehydrogenase, acyl-CoA dehydrogenase, methylcrotonoyl-CoA carboxylase, and enoyl-CoA hydratase. Cycles 2–4: Fatty acid-CoA ligase, hydrolase, and hydroxyacyl-CoA dehydrogenase, yielding a C17-keto intermediate. Final side-chain cleavage generates AD through pyruvate carboxylase and transketolase activity. AD is subsequently dehydrogenated at C1–C2 by 3-ketosteroid Δ^1^-dehydrogenase to form ADD. During the fourth β-oxidation cycle, the intermediate 4-HBC forms via hydrolytic removal of its CoA moiety. Analogous to ADD synthesis, 4-HBC is dehydrogenated by 3-ketosteroid Δ^1^-dehydrogenase to yield 1,4-HBC.

It is worth noting that the C22 double bond of stigmasterol has little effect on early β-oxidation due to its distal position relative to the initial cleavage site. However, as shown in Figure 3 (dark blue shadow), the spatial accessibility of late intermediates (e.g., C20–C22 fragments) near the double bond is reduced due to the presence of residues at both ends of the molecule, which may hinder the enzymatic binding and catalysis in the second β-oxidation cycle.

### 3.5. The Difference in Conversion Rate Was Analyzed by Molecular Docking

**Figure 4:**
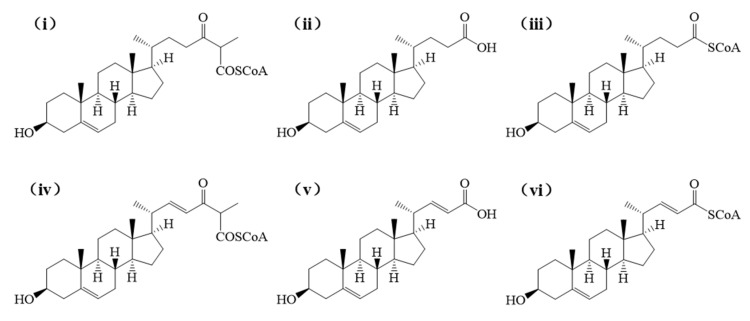
The intermediates obtained from the biotransformation of β-sitosterol and stigmasterol by *Mycobacterium*, respectively. Among them, (**i**–**iii**) denote the intermediates derived from β-sitosterol conversion, whereas (**iv**–**vi**) correspond to those generated during stigmasterol biotransformation.

Figure 3 indicates that during the second cycle of β-oxidation of sterol molecules, the catalytic sites of enzymatic proteins (e.g., hydrolases, fatty acid-CoA ligases, acyl-CoA dehydrogenases) are spatially proximate to the C22 double bond in stigmasterol’s side chain. This proximity likely induces significant steric hindrance, thereby impeding substrate-enzyme interactions and potentially limiting the conversion efficiency of stigmasterol. Given that acyl-CoA synthetase shares functional redundancy with fatty acid-CoA ligase (both exhibit medium-to-long-chain fatty acid-CoA ligase activity) and demonstrates substantially higher expression levels (Table 2), it was prioritized as the primary model for molecular docking simulations. Using AutoDock Vina 1.1.2, we performed molecular docking between these enzymes and sterol-derived small molecules generated during β-sitosterol and stigmasterol bioconversion. The expression levels of docked enzymes are detailed in Table 2, structural formulas of small molecules are shown in Figure 4, and binding energies are summarized in Table 3.

**Table 2:** Enzymes in molecular docking and their circadian expression profiles.

Enzymes	Gene ID	Accession ID	60 h	120 h	Docking Molecules
Hydrolase	ASD37_17210	A0A0T1WAV6	0.83 ± 0.01	0.78 ± 0.01	(i)(iv)
	ASD37_20395	A0A0T1W815	0.34 ± 0.01	0.37 ± 0.10	
Fatty acid-CoA ligase	ASD37_14510	A0A0T1WE13	1.86 ± 0.18	1.74 ± 0.13	(ii)(v)
Acyl-CoA synthase	ASD37_29270	A0A0T1W1C0	32.89 ± 0.10	42.18 ± 0.70	
	ASD37_08775	A0A0T1WFV2	20.52 ± 0.16	25.25 ± 1.65	
Acyl-CoA dehydrogenase	ASD37_29235	A0A0T1W1E3	6.81 ± 0.01	6.87 ± 0.46	(iii)(vi)

**Table 3:** Affinity of enzymes and legends.

	Affinity(kcal/mol)	Affinity (kcal/mol)		Affinity (kcal/mol)	Affinity (kcal/mol)
Mode	A0A0T1WAV6-(i)	A0A0T1WAV6-(iv)	Mode	A0A0T1W815-(i)	A0A0T1W815-(iv)
1	−9.5	−9.7	1	−7.7	−6.3
2	−9.5	−9.6	2	−7.7	−6.3
3	−9.4	−9.3	3	−7.4	−6.2
4	−9.3	−9.3	4	−7.3	−6.2
5	−9.2	−9.2	5	−7.3	−6.1
6	−9.1	−9.2	6	−7.3	−6.1
7	−9.0	−8.9	7	−7.1	−6.0
8	−9.0	−8.8	8	−7.0	−5.9
9	−8.9	−8.6	9	−7.0	−5.9
Mode	A0A0T1WE13-(ii)	A0A0T1WE13-(v)	Mode	A0A0T1W1C0-(ii)	A0A0T1W1C0-(v)
1	−8.7	−9.3	1	−9.0	−7.8
2	−8.7	−9.3	2	−7.5	−7.4
3	−8.0	−8.9	3	−7.4	−7.4
4	−8.0	−8.3	4	−7.2	−7.2
5	−7.1	−8.1	5	−7.1	−7.2
6	−7.1	−8.0	6	−7.0	−6.9
7	−7.0	−7.9	7	−7.0	−6.8
8	−7.0	−7.8	8	−6.9	−6.7
9	−7.0	−7.7	9	−6.8	−6.6
Mode	A0A0T1WFV2-(ii)	A0A0T1WFV2-(v)	Mode	A0A0T1W1E3-(iii)	A0A0T1W1E3-(vi)
1	−8.3	−8.7	1	−10.3	−10.2
2	−7.6	−8.1	2	−10.1	−10.0
3	−7.6	−8.1	3	−10.1	−9.9
4	−7.4	−8.1	4	−9.9	−9.6
5	−7.3	−8.0	5	−9.7	−9.5
6	−6.9	−7.5	6	−9.6	−9.4
7	−6.8	−7.3	7	−9.6	−9.4
8	−6.7	−7.3	8	−9.6	−9.3
9	−6.6	−7.2	9	−9.5	−8.9

As detailed in Table 3, all 12 molecular docking groups exhibited binding energies below −5.0 kcal/mol, indicating strong binding affinity and high complementarity between the selected enzymes and the sterol-derived ligands within their respective catalytic pockets. Notably, two enzyme-ligand pairs showed substantial binding energy differences: Hydrolase (A0A0T1W815) bound to ligands (i) and (iv) with energies of −7.7 kcal/mol and −6.3 kcal/mol, respectively (Δ = 1.4 kcal/mol); Acyl-CoA Synthetase (A0A0T1W1C0) bound to ligands (ii) and (v) with energies of −9.0 kcal/mol and −7.8 kcal/mol (Δ = 1.2 kcal/mol). Furthermore, quantitative proteomics revealed that *Acyl-CoA Synthetase* (A0A0T1W1C0) had significantly higher expression levels than A0A0T1WE13 and A0A0T1WFV2 (fold-change > 2.1, *p* < 0.01). Given its role as the dominant enzyme in sterol side-chain β-oxidation and its superior binding kinetics, A0A0T1W1C0 is identified as the rate-limiting enzyme responsible for the constrained conversion efficiency of stigmasterol, likely due to steric hindrance from stigmasterol’s C22 double bond during catalytic pocket engagement.

**Figure 5:**
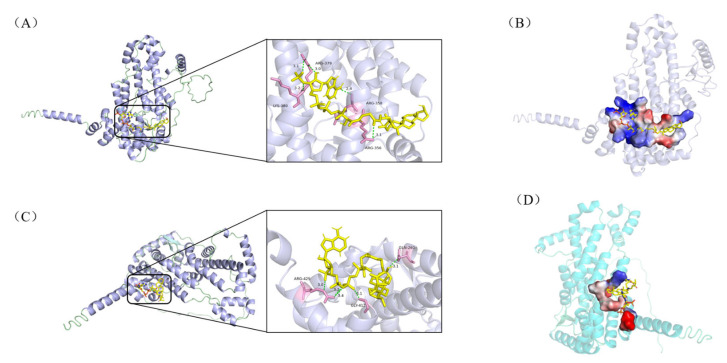
Molecular docking results of hydrolase A0A0T1W815 with intermediates (i) and (iv). The enzyme is shown in cartoon and the ligands as yellow sticks; interacting amino-acid residues are highlighted in pink; hydrogen bonds are indicated by light-green dashed lines. Panel (**A**) illustrates the interaction network between A0A0T1W815 and ligand (i), while panel (**B**) displays the corresponding ligand-binding pocket. Likewise, panel (**C**) depicts the interactions with ligand (iv), and panel (**D**) depicts the topological architecture of its binding pocket.

**Figure 6:**
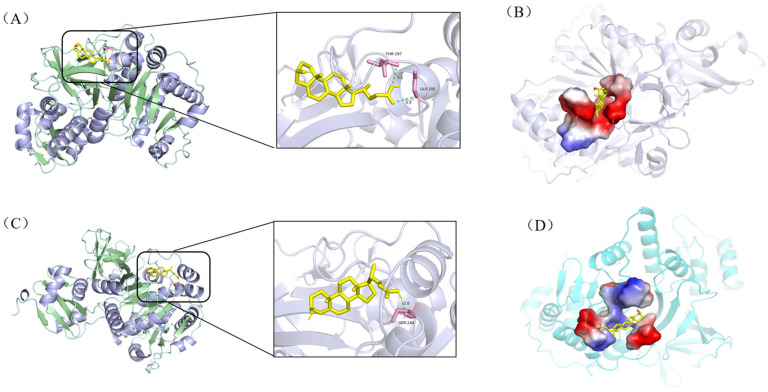
Molecular docking results of acyl-CoA synthetase A0A0T1W1C0 with ligands (ii) and (v). The enzyme is shown in cartoon and the ligands as yellow sticks; pink segments denote amino acid residues forming hydrogen bonds; light-green dashed lines indicate hydrogen bonds. Panel (**A**) illustrates the interaction network between A0A0T1W1C0 and ligand (ii), while (**B**) displays the corresponding ligand-binding pocket. Similarly, (**C**) depicts interactions with ligand (v), and (**D**) visualizes its binding pocket topology.

As illustrated in Figure 5, the hydrogen bonding patterns and spatial geometries between hydrolase A0A0T1W815 and the two ligands (i and iv) exhibited distinct differences. Ligand (i) formed five hydrogen bonds with amino acid residues ARG-379, LYS-380, ARG-356, and ARG-358 (Figure 5A), with bond lengths ranging from 2.4 to 3.3 Å. These interactions provided significant conformational stability to the complex. In contrast, ligand (iv) engaged residues ARG-425, GLY-412, and GLN-260 via five hydrogen bonds (Figure 5C), though with slightly longer bond distances (3.1–3.4 Å), suggesting comparatively weaker electrostatic contributions. Similarly, as shown in Figure 6, the hydrogen bonding patterns and spatial geometries between acyl-CoA synthetase A0A0T1W1C0 and its ligands exhibited distinct variations. Ligand (ii) formed two hydrogen bonds with amino acid residues THR-197 and GLY-195 (Figure 6A), with bond distances of 3.1 Å and 3.3 Å, respectively. In contrast, ligand (v) engaged residue SER-244 via a single hydrogen bond (Figure 6C) characterized by a significantly shorter bond length of 2.0 Å, suggesting stronger electrostatic stabilization at this interaction site.

Collectively, all 12 enzyme-ligand docking groups demonstrated robust binding affinity, as evidenced by binding energies consistently below the threshold of −5.0 kcal/mol. Notably, significant binding energy disparities were observed for hydrolase A0A0T1W815 complexed with ligands (i) and (iv), and acyl-CoA synthetase A0A0T1W1C0 bound to ligands (ii) and (v), suggesting that both enzymes likely serve as rate-limiting factors in the comparatively sluggish bioconversion of stigmasterol. Structural analyses further revealed that while A0A0T1W815 exhibited differential hydrogen bond distances (2.4–3.3Å vs. 3.1–3.4Å) with ligands (i) and (iv), A0A0T1W1C0 showed variations in both bond quantity (two vs. one) and bond length (3.1/3.3Å vs. 2.0Å) with ligands (ii) and (v). These disparities in hydrogen bonding geometry directly modulate intermolecular interaction stability, particularly through suboptimal orbital alignment and desolvation penalties, which manifest macroscopically as differences in catalytic efficiency in the degradation of stigmasterol side chains. Further validation of their restrictive effects through in vitro enzyme activity assays or knockdown/overexpression experiments is needed, which will provide a strong basis for the rational modification of *Mycobacterium*.

## 4. Conclusions

*Mycobacterium* sp. 191574 exhibited markedly divergent conversion rates for different sterols, with β-sitosterol being metabolized significantly faster than stigmasterol. Proteomic mining of sterol metabolic pathways revealed that hydrolase, fatty acid-CoA ligase, and/or acyl-CoA dehydrogenase likely serve as rate-limiting enzymes in stigmasterol bioconversion. Molecular docking simulations further identified hydrolase A0A0T1W815 and acyl-CoA synthetase A0A0T1W1C0 as the primary bottlenecks. Comparative analysis demonstrated distinct spatial geometries and hydrogen bonding patterns between the two enzymes and sterol-derived ligands: β-sitosterol complexes formed more numerous and shorter hydrogen bonds (≤3.3Å) with optimized orbital alignment, whereas stigmasterol interactions exhibited longer bond distances (≥3.1Å) and suboptimal binding poses due to steric hindrance from its C22 double bond. These molecular-scale disparities in docking affinity directly explain the macroscopic divergence in conversion kinetics, providing a mechanistic framework for understanding sterol metabolism and guiding future engineering of high-efficiency Mycobacterium cell factories.

## Data Availability

The original contributions presented in the study are included in the article and Supplementary Materials, and further inquiries can be directed to the corresponding authors.

## Supplementary Materials

The following supporting information can be downloaded at https://www.mdpi.com/article/10.3390/biom15111496/s1, Figure S1. Structural formula of main steroid drug intermediates. Table S1. Enzymes of sterol conversion identified by the proteomics and expression level.

## Abbreviations

AD: androstenedione; ADD: androst-1,4-diene-3,17-dione; 4-HBC: 22-hydroxy-23,24-bisnorchol-4-ene-3-one; 1,4-HBC: 22-Hydroxy-23,24-bisnorchol-1,4-diene-3-one; 9α-OH-AD: 9-hydroxy-4-androstene-3,17-dione; HP-β-CD: 2-Hydroxypropyl-β-cyclodextrin; KstD: 3-ketosteroid-Δ^1^-dehydrogenase; 3α-HSD: 3α-hydroxysteroid dehydrogenase.
