# Supplementary material for: Divergent Conversion Efficiencies of Mycobacterium sp. 191574 for Various Phytosterols and Their Underlying Mechanisms
Source: Biomolecules. 2025 Oct 23;15(11):1496. doi: 10.3390/biom15111496 (PMC12650333; doi:10.3390/biom15111496)
Supplement: Supplementary file 1 [file biomolecules-15-01496-s001.zip › biomolecules-3907312-supplementary.pdf]

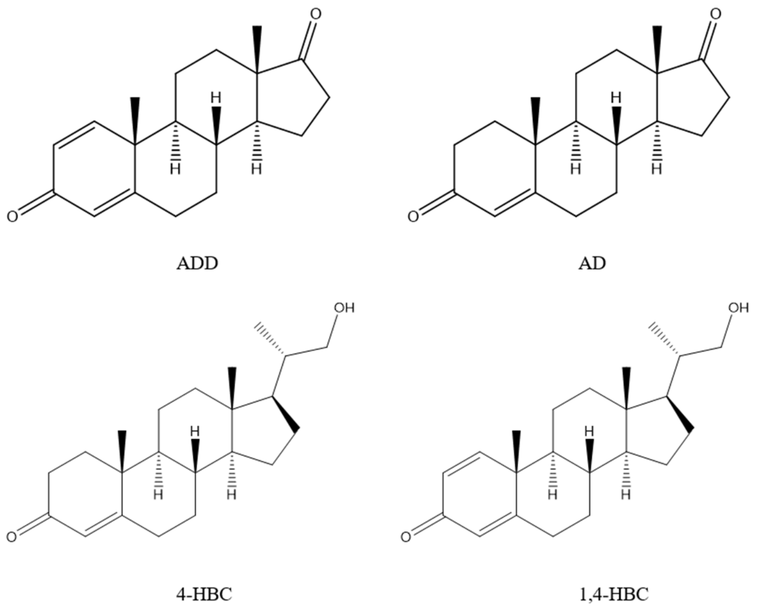

Figure S1. Structural formula of main steroid drug intermediates

Table S1. Enzymes of sterol conversion identified by the proteomics and expression level

Enzymes	Gene ID	Expression level（TPM）	
		60 h	120 h
3-Alpha-hydroxysteroid dehydrogenase	purH	13.76±1.51b	15.29±0.32a
Cytochrome P450	ASD37_25600	0.91±0.17a	0.82±0.04a
Steroid C27-monooxygenase	ASD37_06105	0.51±0.01a	0.62±0.05a
Monooxygenase	groES	14.06±0.38b	15.33±0.67a
	sigA	1.90±0.09a	1.89±0.11a
	ASD37_20840	1.24±0.10a	1.39±0.02a
	ASD37_12895	0.63±0.05a	0.67±0.02a
	ASD37_13430	0.62±0.13a	0.63±0.11a
	ASD37_22545	0.45±0.06a	0.62±0.03b
Acyl carrier protein	ASD37_27505	78.82±14.91a	61.67±3.82b
Acetyl-coenzyme A synthetase	rpsS	59.08±5.95a	51.47±2.68b
Acetyltransferase	ASD37_00560	5.36±0.43a	5.38±0.01a
	ASD37_05675	0.89±0.01a	1.26±0.02a
Acyl-CoA synthetase	rpoA	3.88±0.01a	3.99±0.03a
	ASD37_20425	3.01±0.34a	1.94±0.04b
	ASD37_12405	1.68±0.24a	1.86±0.07a
	ASD37_03515	0.73±0.06b	1.01±0.05a

	polA	0.89±0.17a	0.93±0.11a
	ASD37_20515	0.62±0.11a	0.63±0.12a
	menB	0.59±0.08b	0.73±0.01a
Acyl-CoA dehydrogenase	rpoC	14.55±0.23a	14.18±0.64a
	ASD37_29615	4.14±0.28a	4.6±0.26a
	ASD37_09190	6.02±2.05a	5.13±0.54b
	ASD37_29630	2.11±0.16a	2.59±0.54a
	dapB	2.09±0.15a	2.47±0.22a
	rplN	3.10±0.10a	2.85±0.21a
	kgd	2.02±0.03a	2.13±0.15a
	dnaA	1.46±0.23a	1.33±0.12a
	pyrG	1.40±0.17a	1.33±0.04a
	uvrA	2.05±0.27a	1.41±0.12a
	ASD37_29030	1.54±0.21a	1.16±0.19a
	ASD37_17160	0.82±0.07a	0.84±0.03a

Table S1. Enzymes of sterol conversion identified by the proteomics and expression level (continue)

Enzymes	Gene ID	Expression level （TPM）	
		60 h	20 h
Acyl-CoA dehydrogenase	guaA	0.58±0.05[a]	0.75±0.01[a]
	ASD37_04050	0.52±0.14[a]	0.83±0.08[a]
	hemL	0.63±0.14[a]	0.61±0.06[a]
	ASD37_09015	0.52±0.06[a]	0.58±0.06[a]
Enoyl-CoA hydratase	ASD37_17430	5.23±0.13[b]	6.24±0.18[a]
	ASD37_12925	3.3±0.17[a]	4.55±0.36[a]
	ASD37_28525	4.27±0.30[a]	3.02±0.09[b]
	ASD37_06020	1.47±0.02[b]	1.72±0.05[a]
	ASD37_25400	1.77±0.05[a]	1.58±0.12[a]
	pdxS	0.96±0.11[a]	0.82±0.09[a]
	ASD37_22120	0.35±0.01[a]	0.66±0.07[a]
	rpsT	0.75±0.16[a]	0.59±0.02[a]
	glgC	0.33±0.07[a]	0.56±0.07[a]
Oxidoreductase	ASD37_00505	20.5±2.73[a]	21.05±0.96[a]
	rpsA	6.24±0.22[a]	5.59±0.07[a]
	ASD37_28760	3.30±0.23[b]	4.66±0.37[a]
	ASD37_07670	0.93±0.12[a]	1.05±0.13[a]
	purC	0.80±0.01[a]	0.81±0.20[a]
Short-chain dehydrogenase	ASD37_25340	11.81±1.35[b]	17.19±0.02[a]
	ASD37_06165	3.47±1.55[a]	2.47±0.13[a]
	whiA	0.97±0.02[a]	1.38±0.07[a]
	asd	0.77±0.10[a]	0.85±0.05[a]
	ASD37_20795	0.54±0.02[a]	0.77±0.01[a]
	ASD37_18905	0.79±0.08[a]	0.76±0.11[a]
	ASD37_16505	0.53±0.06[a]	0.68±0.02[a]

Acyl-CoA thioesterase II	ASD37_16600	2.69±0.50[b]	8.06±0.09[a]
Pyruvate carboxylase	ASD37_29385	5.61±0.97[a]	5.87±0.20[a]
Transketolase	rpsF	9.17±0.08[a]	8.36±0.94[a]
3-Ketosteroid-delta-1-dehydrogenase	leuS	0.64±0.02[a]	0.67±0.01[a]
3-Ketosteroid-9-alpha-hydroxylase	ASD37_27185	0.60±0.11[a]	0.58±0.06[a]
